# 1*α*,25-dihydroxyvitamin D3 in combination with transforming growth factor-*β* increases the frequency of Foxp3^+^ regulatory T cells through preferential expansion and usage of interleukin-2

**DOI:** 10.1111/imm.12289

**Published:** 2014-07-29

**Authors:** Emma S Chambers, Duangchan Suwannasaen, Elizabeth H Mann, Zoe Urry, David F Richards, Ganjana Lertmemongkolchai, Catherine M Hawrylowicz

**Affiliations:** 1 MRC and Asthma-UK Centre for Allergic Mechanisms in Asthma, King's College LondonLondon, UK; 2 Cellular and Molecular Immunology Unit, Faculty of Associated Medical Sciences, Centre for Research and Development of Medical Diagnostic Laboratories (CMDL), Khon Kaen UniversityKhon Kaen, Thailand

**Keywords:** 1*α*,25-dihydroxyvitamin D3, Foxp3^+^ regulatory T cells, interleukin-2, transforming growth factor-*β*

## Abstract

A high prevalence of vitamin D insufficiency and deficiency exists worldwide, which is associated with an increased incidence and severity of a range of immune-mediated diseases. This has resulted in considerable interest in the immunodulatory functions of vitamin D. The active form of vitamin D, 1*α*,25-dihydroxyvitamin D3 [1,25(OH)_2_D3], has been shown to increase the frequency of Foxp3^+^ CD4^+^ T regulatory (Treg) cells when present at high concentrations or under strong T-cell stimulation in culture. Supporting evidence exists *in vivo* for a positive association between serum 25(OH)D and Foxp3^+^ Treg cell numbers in humans. The aim of this work was to identify the cytokine milieu required *in vitro* to promote Foxp3^+^ Treg cells in cultures containing 1,25(OH)_2_D3 at more moderate concentrations (10^−7 ^m). Stimulation of human CD4^+^ T cells with a combination of 1,25(OH)_2_D3 and transforming growth factor-*β* (TGF-*β*) greatly increased the frequency of Foxp3^+^ Treg cells, which is proposed to result from the preferential expansion of Foxp3^+^ Treg cells, as compared with the Foxp3^−^ effector T cells, in culture. The differential effect on proliferation may result from enhanced availability and usage of interleukin-2 by the Foxp3^+^ Treg cells compared with Foxp3^−^ effector T cells. In summary, modulation of the cytokine environment to one high in TGF-*β* in the presence of 1,25(OH)_2_D3 (10^−7^ m) significantly increased Foxp3^+^ Treg cell frequency. These data provide additional evidence for the important immunomodulatory properties of 1,25(OH)_2_D3 that exist and may help to control inflammatory diseases.

## Introduction

A high prevalence of vitamin D insufficiency has been widely linked with a number of immune-mediated diseases including autoimmune conditions and asthma.^1^ This association is believed to be due, in part, to the numerous immunomodulatory properties of the active form of vitamin D, 1*α*,25-dihydroxyvitamin D3 [1,25(OH)_2_D3]. 1,25(OH)_2_D3 has been reported to inhibit proliferation of effector cells and inflammatory cytokines while enhancing production of anti-inflammatory cytokines such as interleukin-10 (IL-10) and to increase regulatory T (Treg) cell populations.^2^,^3^

Evidence that 1,25(OH)_2_D3 may be important in the induction and maintenance of Foxp3^+^ Treg cells *in vivo* derives from data showing that serum concentrations of 25-hydroxyvitamin D [25(OH)D] positively correlates with the number and frequency of Foxp3^+^ Treg cells in the peripheral blood as well as with the frequency of Foxp3^+^ Treg cells in bronchoalveolar lavage fluid of paediatric asthma patients.^4^–^7^ This is supported by reports using animal models and also by *in vitro* studies with human peripheral blood T cells.^8^–^10^ We have previously reported that 1,25(OH)_2_D3 increases the frequency of Foxp3^+^ Treg cells as well as IL-10^+^ Treg cells *in vitro*, although little or no co-expression of these molecules was observed in culture.^5^,^11^ However, only high doses of vitamin D, in the order of 10^−6^ m 1,25(OH)_2_D3, and relatively long culture periods of 2 weeks, led to the reproducible increase in the frequency of Foxp3^+^ Treg cells, with lower concentrations of 1,25(OH)_2_D3 (10^−7^–10^−8^ m) more likely to increase IL-10^+^ Treg cells.^5^

The cytokine milieu undoubtedly plays an important role in Foxp3^+^ Treg cell generation. For example, in our 1,25(OH)_2_D3 culture system the frequency of Foxp3^+^ Treg cells was reduced by the addition of IL-10 into the culture but was increased by anti-IL-10 receptor antibody. Transforming growth factor-*β* (TGF-*β*) has been widely implicated in the generation of Foxp3^+^ Treg cells from naive CD4^+^ T cells.^12^–^14^ Additionally TGF-*β* in combination with the vitamin A metabolite, retinoic acid, is capable of converting effector cells into Foxp3^+^ Treg cells with gut homing properties, facilitated by mucosal CD103^+^ dendritic cells.^15^–^20^ To maintain stable Foxp3 expression, TGF-*β* is required to bind to a conserved non-coding sequence region upstream of the *Foxp3* gene.^21^

Another cytokine important for the survival, maintenance and proliferation of Foxp3^+^ Treg cells is IL-2.^22^ Although IL-2 was originally described as a T-cell growth factor, IL-2 knockout mice were shown to develop a lethal lymphoproliferative disease as a result of the lack of Treg cells.^23^–^25^ Foxp3^+^ Treg cells express high levels of CD25, the high-affinity subunit of the IL-2 receptor, and high levels of IL-2 are required for expansion of Foxp3^+^ Treg cells in culture.^26^–^30^ Additionally it has been shown that IL-2 inhibits the generation of T helper type 17 cells as well as the production of IL-17A, which are known to be inhibitory to Foxp3^+^ Treg cell development.^31^ The immunomodulatory properties of IL-2 have led to it being proposed to have therapeutic potential in diseases such as graft-versus-host disease.^32^ Interestingly, although Foxp3^+^ Treg cells are dependent upon IL-2, they appear incapable of producing IL-2 themselves and are dependent on IL-2 production from effector T cells.^33^

The aim of this work was to identify which cytokine environment was necessary to increase the frequency of Foxp3^+^ Treg cells in the presence of lower, putatively more physiological concentrations of 1,25(OH)_2_D3. We hypothesized that lower concentrations of 1,25(OH)_2_D3 in an environment high in TGF-*β* would increase the frequency of Foxp3^+^ Treg cells. To understand the mechanisms behind this, the impact of TGF-*β* on the proliferation, CD25 expression, IL-2 synthesis and signal transducer and activator of transcription 5 (STAT5) phosphorylation of CD4^+^ Foxp3^+^ and CD4^+^ Foxp3^−^ populations was compared. The data suggest that preferential survival and expansion of Foxp3^+^ Treg cells occurs through enhanced CD25 expression and greater IL-2 consumption, as determined by phosphorylation of STAT5.

## Materials and methods

### Cell isolation and culture

Peripheral blood was obtained from healthy donors after receiving the approval of the Guy's Hospital Ethics Committee (09/H0804/77) and full written informed consent from all subjects. CD4^+^ T cells were purified from peripheral blood mononuclear cells by positive selection using Dynabeads (Invitrogen, Paisley, UK) as previously described.^5^ Cells (1 × 10^6^ cells/ml) were cultured in RPMI-1640 containing 10% fetal calf serum, 2 mm l-glutamine and 50 μg/ml gentamycin, and stimulated with plate-bound anti-CD3 (1 μg/ml; OKT-3) plus 50 IU/ml recombinant human IL-2 (Eurocetus, Harefield, UK), in the presence or absence of 1,25(OH)_2_D3 (ENZO Life Sciences, Exeter, UK), TGF-*β* and/or blocking anti-IL-10 receptor antibody (R&D Systems, Abingdon, UK) at the indicated concentrations. For Treg cell and effector T cell isolation, CD4^+^ cells were isolated by negative selection using the Rosette CD4^+^ enrichment kit (StemCell Technologies, Grenoble, France) from cones obtained from the National Blood Service. To identify ‘Treg’ CD4^+^ T cells (CD25^+^ CD127^lo^) and ‘effector’ CD4^+^ T cells (CD25^–^ CD127^hi^) isolation was performed using a FACSAria Flow Cytometer (BD Biosciences, Oxford, UK) and sort criteria were based on CD127 and CD25 surface staining as described previously.^5^

Cell proliferation was studied by labelling populations with CellTrace Violet (Invitrogen). Proliferation was assessed as the loss of CellTrace™ Violet fluorescence on day 7 and day 14 cell cultures using a FACSCanto (BD Biosciences).

### Flow cytometry

CD3, CD25 (SK7 and M-A251 respectively; BD Biosciences) and CD127 (eBioRDR5; eBiosciences Hatfield, UK) antibodies were used for cell surface phenotyping. Cells were then further stained for intranuclear Foxp3 (PCH101; eBiosciences) using the Foxp3 staining kit as per the manufacturer's instructions (eBiosciences).

For intracellular cytokine staining on day 7, cells were restimulated for 4 hr with 5 ng/ml PMA and 500 ng/ml ionomycin, with 2 μm monensin (Sigma-Aldrich, St Louis, MO) added for the final 2 hr. Cells were washed, fixed and permeabilized using Cytofix/Cytoperm kit (BD Biosciences) and then stained with fluorescently labelled monoclonal antibodies to IL-2 (MQ1-17H12; eBiosciences). Dead cells (7-aminoactinomycin D-positive; Sigma-Aldrich, Gillingham, UK) were gated out.

For phospho-STAT5 staining two different buffer kits were used with the same antibody clone: 47/Stat5[pY694] obtained from BD Biosciences. The buffer set used for phospho-STAT5 staining on total CD4^+^ cell populations was PerFix EXPOSE Kit (Beckman Coulter, High Wycombe, UK). The buffer set used for phopspho-STAT5 staining on sorted Treg cell and effector T cell populations was CytoFix/CytoPerm Buffer and Perm Buffer III (BD Biosciences). Both buffer sets were used as per the manufacturer's instructions.

### Quantitative RT-PCR

RNA was extracted from cell pellets using the RNeasy Mini kit (Qiagen, Crawley, UK) according to the manufacturer's instructions. The RNA was quantified using a Nanodrop ND-1000 spectrophotometer (ThermoScientific, Wilmington, DE) then 250 ng of RNA was reverse transcribed into cDNA. Quantitative RT-PCR was performed in triplicate using an Applied Biosystems 7900 HT system and FAM-labelled Assay-on-Demand reagent sets for *foxp3* (Hs00203958_m1) and *il2* (Hs00174114_m1). Quantitative RT-PCR were multiplexed using VIC-labelled 18S primers and probes (Hs99999901_s1) as an endogenous control and analysed using SDS software version 2.1 (Applied Biosystems, Foster City, CA), according to the 2^−(ΔΔCt)^ method.

### Flow cytometric and statistical analysis

Flow data obtained from BD FACSCalibur were analysed using CellQuest Pro (BD Biosciences) while samples acquired on a BD FACSCanto or BD LSR Fortessa were analysed using FlowJo (Treestar Inc., Ashland, OR). Data analysis was performed in Graphpad Prism version 6.00 for Windows obtained from Graphpad Software Inc. (San Diego, CA) using statistical tests detailed in the figure legend.

## Results

### TGF-*β* increased the frequency of 1,25(OH)_2_D3 induced Foxp3^**+**^ Treg cells

**Figure 1:**
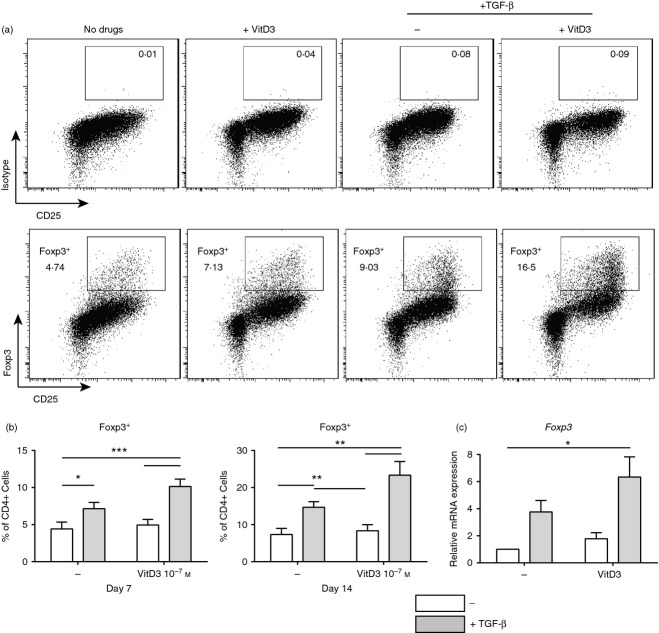
1,25(OH)_2_D3 in the presence of transforming growth factor-*β* (TGF-*β*) increases the frequency of Foxp3^+^ regulatory T cells. CD4^+^ T cells stimulated with anti-CD3 and interleukin-2 (IL-2) for 7 or 14 days alone (No drugs; −) or additionally with 1,25(OH)_2_D3 (VitD3; 10^−7^ m)and/or TGF-*β* (2 ng/ml). (a) Representative dot plots; (b) cumulative data of Foxp3^+^ expression in CD4^+^ cells at day 7 (left; *n *=* *7) and day 14 (right; *n *=* *5); (c) cumulative data of relative *foxp3* gene expression as determined by quantitative RT-PCR in the presence or absence of TGF-*β* (grey; *n *=* *5).Values represent the percentage of gated live cells in each gate. (b) Assessed by repeated measures one-way analysis of variance with Tukey's multiple comparison test (c) assessed by repeated measures one-way analysis of variance with Freidman's post-test. *P ≤* *0·05, **P ≤* *0·01, ***P ≤* *0·001.

We previously reported that human CD4^+^ T cells stimulated with anti-CD3 (1 μg/ml) and IL-2 (50 IU/ml) in the presence 10^−7^ m 1,25(OH)_2_D3 did not significantly increase the frequency of Foxp3^+^ Treg cells in culture. In the current study the addition of TGF-*β* was assessed in combination with 1,25(OH)_2_D3, to determine whether it could promote Foxp3^+^ Treg cell generation *in vitro*. The addition of TGF-*β* into CD4^+^ T-cell cultures containing 10^−7^ m 1,25(OH)_2_D3 led to a reproducible and significant increase in Foxp3^+^ CD25^hi^ Treg cells after both 7 and 14 days of culture (Fig. 1a,b). This increase in Foxp3^+^ Treg cell populations was not due to differential cell death (see Supporting information, Fig. S1A). Quantitative RT-PCR analysis demonstrated a significant increase in *foxp3* gene expression in these conditions (Fig. 1c). The combination of TGF-*β* and the blocking antibody to IL-10 receptor was assessed based on our earlier findings,^5^ in the same experimental system. Although the combination of TGF-*β* and anti-IL-10 receptor significantly increased the frequency of Foxp3^+^ Treg cells (see Supporting information, Fig. S2), this increase was not significantly greater than the effects of TGF-*β* alone. Therefore the rest of this study focused on the mechanisms through which TGF-*β*, in combination with 10^−7^ m 1,25(OH)_2_D3, promotes Foxp3^+^ Treg cell frequency.

### TGF-*β* with 1,25(OH)_2_D3 lead to preferential expansion of Foxp3^**+**^ Treg cells

**Figure 2:**
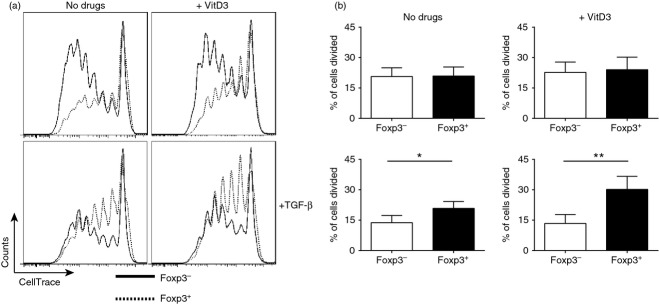
Increased proliferation in Foxp3^+^ population compared with the Foxp3^−^ population in conditions that enhance Foxp3^+^ Treg cells. CD4^+^ T cells stimulated for 7 days with anti-CD3 and interleukin-2 (IL-2) alone (No drugs; −) or additionally with 1,25(OH)_2_D3 (VitD3; 10^−7^ m) and/or transforming growth factor-*β* (TGF*β*; 2 ng/ml). Cells were labelled at day 0 with CellTrace™ and proliferation was assessed by loss in fluorescence. (a) Representative histograms of CellTrace expression in Foxp3^−^ (black; solid) and Foxp3^+^ (black; dotted) CD4^+^ T cells. (b) Cumulative data of the percentage of original population divided in Foxp3^−^ (white) and Foxp3^+^ (black) at day 7 (*n *=* *5). (b) Assessed by paired *t*-test. *P ≤* *0·05, **P ≤* *0·01.

To investigate the mechanism through which the combination of 10^−7^ m 1,25(OH)_2_D3 and TGF-*β* increases the frequency of Foxp3^+^ Treg cells, proliferation was assessed in CD4^+^ T-cell populations. Cells were labelled at day 0 with CellTrace™ and then proliferation was assessed by loss of fluorescence after 7 days of culture. Foxp3 staining was additionally performed to determine the impact of these culture conditions on the proliferation of Treg (Foxp3^+^) versus effector (Foxp3^−^) CD4^+^ T cells (Fig. 2a). Significantly more proliferation, expressed as per cent of divided cells, was seen in the Foxp3^+^ compared with the Foxp3^−^ T-cell population in cultures containing 1,25(OH)_2_D3 plus TGF-*β*, suggesting that these conditions favour the expansion of Foxp3^+^ Treg cells (Fig. 2a,b). This effect of 1,25(OH)_2_D3 and TGF-*β* on the proliferation of effector T cells may explain why there was differential cell recovery at day 7 (see Supporting information, Fig. S1B).

### 1,25(OH)_2_D3 plus TGF-*β* significantly increased IL-2 expression in CD4^**+**^ T cells

Since the addition of TGF-*β* induced preferential expansion of Foxp3^+^ Treg cells in 1,25(OH)_2_D3-containing cultures, how TGF-*β* modulates Foxp3^+^ Treg cells in this experimental system was addressed next. Interleukin-2 is important for induction, maintenance and proliferation of Foxp3^+^ Treg cells and the impact of TGF-*β* on IL-2 expression and signalling was therefore investigated.^13^,^22^,^26^–^28^,^34^

**Figure 3:**
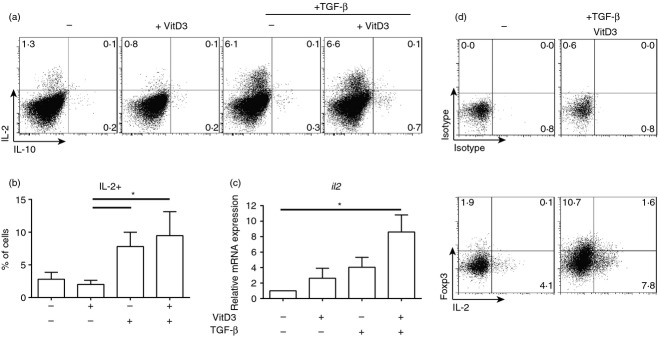
Increased interleukin-2 (IL-2) secretion in conditions that enhance the frequency of Foxp3^+^ regulatory T (Treg) cells. CD4^+^ T cells stimulated for 7 days with anti-CD3 and IL-2 alone (No drugs; −) or additionally with 1,25(OH)_2_D3 (VitD3; 10^−7^ m) and/or transforming growth factor-*β* (TGF-*β*; 2 ng/ml). Intracellular cytokine staining was performed following 4 hr of stimulation with PMA/ionomycin with Monensin added for the final 2 hr. (a) Representative dot plots, (b) cumulative data of frequency of IL-2^+^ cells at day 7 (*n *=* *5), (c) cumulative data of relative *il2* gene expression as determined by quantitative RT-PCR (*n *=* *7), (d) representative dot plots of Foxp3 and IL-2 co-staining. Values represent the percentage of gated live cells in each quadrant. (b) and (c) assessed by repeated measures one-way analysis of variance with Freidman's post-test.*P ≤* *0·05.

Interleukin-2 production was assessed by intracellular cytokine staining to determine whether the conditions that increased the frequency of Foxp3^+^ Treg cells also increased IL-2 production. Although the presence of 10^−7^ m 1,25(OH)_2_D3 in culture showed a trend to decreased IL-2 in culture (Fig. 3a), TGF-*β* alone and in combination with 10^−7^ m 1,25(OH)_2_D3 significantly increased the frequency of IL-2^+^ CD4^+^ T cells compared with 1,25(OH)_2_D3 alone (Fig. 3a,b). Furthermore, *il2* mRNA expression was significantly increased in cultures containing 10^−7^ m 1,25(OH)_2_D3 and TGF-*β* compared with the no-drug condition (Fig. 3c). Intracellular co-staining of IL-2 and Foxp3 showed that there was little to no co-expression of IL-2 and Foxp3 (Fig. 3d), suggesting that IL-2 is being produced by Foxp3^−^ effector T cells.

These data suggest that one potential mechanism through which the combination of 1,25(OH)_2_D3 and TGF-*β* enhance the expansion of Foxp3^+^ Treg cells is by increasing the availability of IL-2 in culture.

### Modulation of CD25 expression in cultures containing 1,25(OH)_2_D3 and TGF-*β*

**Figure 4:**
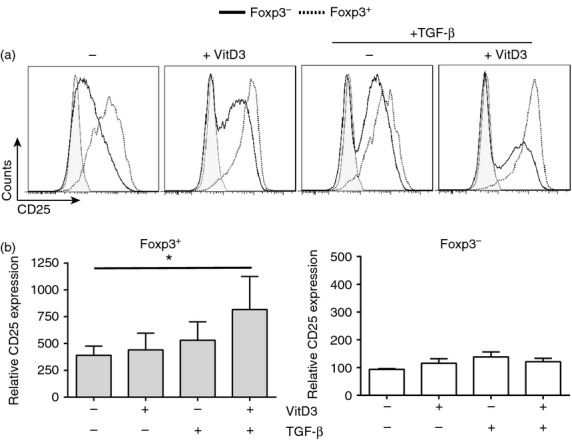
CD25 expression decreased on Foxp3^−^ but not Foxp3^+^ CD4^+^ T cells in cultures containing transforming growth factor-*β* (TGF*β*) and 1,25(OH)_2_D3 (10^−7^ m). Total CD4^+^ T cells were cultured for 7 days with anti-CD3 and interleukin-2 (IL-2) alone (No drugs; −) or additionally with 1,25(OH)_2_D3 (VitD3; 10^−7^ m) and/or TGF-*β* (2 ng/ml). (a) Representative histograms of CD25 expression in Foxp3^−^ (black; solid) and Foxp3^+^ (black; dotted) CD4^+^ T cells compared with isotype control (grey; shaded); (b) cumulative data of relative CD25 expression (compared with total CD25 expression in the no drug Foxp3^−^ cells) on Foxp3^+^ or Foxp3^−^ cells CD4^+^ T cells (*n *=* *4). (b) Assessed by repeated measures one-way analysis of variance with Freidman's post-test. *P ≤* *0·05.

Expression of the high affinity IL-2 receptor *α* (IL-2R*α*; CD25), was next assessed on Foxp3^+^ and Foxp3^−^ CD4^+^ T cells. Following culture of CD4^+^ T cells for 7 days with anti-CD3 and IL-2 plus 10^−7^ m 1,25(OH)_2_D3 and TGF-*β*, a significant increase in the expression of CD25 on Foxp3^+^ Treg cells, but not on Foxp3^−^ CD4^+^ T cells was observed compared with cultures lacking 1,25(OH)_2_D3 and TGF-*β* (Fig. 4a,b). These data suggest that there is a greater capacity for IL-2 consumption by Foxp3^+^ Treg cells under these culture conditions.

**Figure 5:**
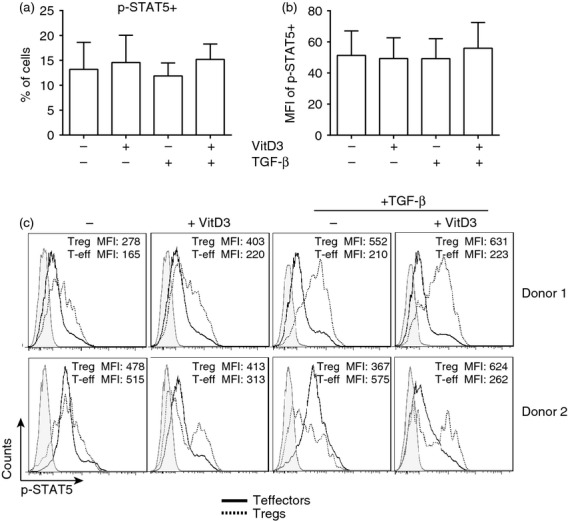
Interleukin-2 (IL-2) is preferentially consumed by Foxp3^+^ regulatory T (Treg) cells when transforming growth factor-*β* (TGF-*β*) and 1,25(OH)_2_D3 are present. Total CD4^+^ T cells were cultured for 7 days with anti-CD3 and IL-2 (No drugs; −) or additionally with 1,25(OH)_2_D3 (VitD3; 10^−7^ m) and/or TGF-*β* (2 ng/ml). At day 7 cells were stained for phosphorylation of signal transducer and activator of transcription 5 (p-STAT5; Y694). (a) Cumulative data showing the frequency of p-STAT5^+^ and (b) the mean fluorescence intensity (MFI) of the p-STAT5^+^ cells (*n *=* *4). (c) Subsequently CD4^+^ T cells were isolated by negative selection then further sorted based on CD25 and CD127 cell surface staining for Treg cells (CD127^lo^ CD25^hi^) and effector T cells (CD127^hi^ CD25^lo^) (see Supporting information, Fig. S2). Either Treg cells or effector T cells were labelled with CellTrace Violet then cultured at the ratio 1: 9 (Treg : T effector) for 7 days with anti-CD3 and IL-2 alone (No drugs; −), or additionally with 1,25(OH)_2_D3 (VitD3; 10^−7^ m) and/or TGF-*β* (2 ng/ml). Representative histograms showing p-STAT5 staining in effector T cells (black; solid), Treg cells (black; dotted) and unstained (grey), with accompanying MFI for the effector T cells (Teff) and T reg cells.

To prove that IL-2 was being predominantly consumed by Foxp3^+^ Treg cells in the presence of 10^−7^ m 1,25(OH)_2_D3 and TGF-*β*, phosphorylation of STAT5 was used as a marker of IL-2 signalling. Downstream of IL-2 ligation of CD25 there is phosphorylation of tyrosine at site 694 (Y694), a prerequisite for STAT5 activation.^35^,^36^ When phosphorylation of STAT5 was assessed in total CD4^+^ T-cell cultures no significant difference was observed between the culture conditions (Fig. 5a,b). To understand further which cells were using the IL-2 produced in the presence of 1,25(OH)_2_D3 and TGF-*β* the consumption of IL-2 by Foxp3^+^ Treg cells was assessed. As there is incompatibility between buffers, Foxp3 and STAT5 staining could not be performed simultaneously. Therefore Treg cells were sorted based on CD127^lo^ CD25^hi^ expression, and effector T cells were identified as being CD127^hi^ CD25^lo/–^. Typically, 87% of the Treg cells isolated were Foxp3^+^, whereas < 1% of the effector T cells were Foxp3^+^ (see reference 5). The Treg or effector T cells were labelled with CellTrace™ and co-cultured at a regulator : effector ratio of 1 : 9 with anti-CD3 and IL-2 in the presence or absence of 10^−7^ m 1,25(OH)_2_D3 and/or TGF-*β*. At day 7 intracellular staining for STAT5 phosphorylation was performed. In cultures containing 1,25(OH)_2_D3 and TGF-*β*, phosphorylation of STAT5 was considerably higher in Treg cells and lower in effector T cells compared with the no drug and 1,25(OH)_2_D3 conditions (Fig. 5c).

In conclusion, these data suggest that 10^−7^ m 1,25(OH)_2_D3 and TGF-*β* collectively promote Foxp3^+^ Treg cells through increasing IL-2 secretion from CD4^+^ effector T cells, which is then preferentially consumed by Foxp3^+^ Treg cells.

## Discussion

The cytokine environment is recognized to play an important role in the generation and maintenance of Foxp3^+^ CD4^+^ T-cell populations. Evidence from epidemiological studies, animal models and *ex vivo* data all suggest a role for vitamin D in positively influencing the frequency of CD4^+^ Foxp3^+^ Treg cells.^4^–^7^ Studies with human T cells suggest that *in vitro* 1,25(OH)_2_D3 optimally enhances the frequency of these cells only when present at very high concentrations or under conditions of strong T-cell stimulation.^5^,^8^ The data presented in this paper propose that the cytokines IL-2 and TGF-*β* have key co-operative functions in enhancing frequencies of 1,25(OH)_2_D3-induced Foxp3^+^ Treg cells. These data complement our earlier findings, which demonstrated that the cytokine milieu, in particular IL-10, had a negative impact on the frequency of 1,25(OH)_2_D3-induced Foxp3^+^ Treg cells and enhanced IL-10^+^ Treg cells.^5^

Historically, TGF-*β* has been shown to be important for the induction of Foxp3^+^ Treg cells from naive CD4^+^ T cells, and IL-2 is proposed to be an essential component in TGF-*β*-mediated induction of Foxp3^+^ Treg cells.^13^,^30^,^34^ Additionally, TGF-*β* has the capacity to convert effector as well as naive CD4^+^ T cells into Foxp3^+^ Treg cells when in the presence of the vitamin A metabolite retinoic acid.^15^–^20^ The current work builds on these earlier findings and proposes that like retinoic acid, the combination of TGF-*β* and 1,25(OH)_2_D3 significantly increase the frequency of Foxp3^+^ Treg cells.

A proposed mechanism for the increase in Foxp3^+^ T cells in cultures containing TGF-*β* and 1,25(OH)_2_D3 was due to the preferential expansion of these cells compared with the Foxp3^−^ CD4^+^ T cells. This preferential expansion of Foxp3^+^ Treg cells is believed to be due, at least in part, to an increase in IL-2 production in culture in the presence of TGF-*β* alone or in combination with 1,25(OH)_2_D3. Interleukin-2 has been shown to be vitally important for the TGF-*β*-mediated increase in Foxp3^+^ Treg cells, and in the absence of IL-2 there was no Foxp3^+^ Treg cell induction.^13^,^30^,^34^ As predicted, the increased production of IL-2 in conditions containing TGF-*β* originated from the effector T cells, which is in agreement with previous reports describing that Foxp3^+^ Treg cells do not produce their own IL-2. Interestingly unpublished data from our laboratory showed that in the absence of Treg cells there was more proliferation in the effector T cell cultures, again indicating that the Foxp3^+^ Treg cells were out-competing effector T cells in culture for the growth factor IL-2. Furthermore, in the presence of both 1,25(OH)_2_D3 and TGF-*β* there was enhanced usage of IL-2, as evidenced by the increased phosphorylation of STAT5 in Foxp3^+^, compared with the Foxp3^−^ T-cell population.

The study of human Foxp3^+^ Treg cell populations is difficult because the immunological tools do not readily exist to adequately distinguish between ‘natural’ Foxp3^+^ Treg cells, adaptive/induced Treg cell or even early activation-dependent Foxp3 expression. However evidence from our earlier studies,^5^ where we observed that 1,25(OH)_2_D3 caused little to no inhibition of the proliferation of sorted populations of Foxp3^+^ Treg cells (CD25^hi^ CD127^lo^), but significantly inhibited the proliferation of effector T cells suggests that the observed effects are due to effects on existing rather than *de novo* induced Treg cells. It also seems unlikely that we are observing proliferation of activation-induced Foxp3^+^ cells because our culture conditions (anti-CD3 and IL-2) do not provide a strong enough stimulus. Furthermore, the cultures in this study have a much longer duration whereas activation-induced Foxp3 expression is a rapid event which is then lost.^37^ Additional evidence to support our data includes the strong positive correlation observed *in vivo* between vitamin D sufficiency and Foxp3^+^ Treg cell numbers and frequency.^4^,^5^,^7^

Early studies into the effects of 1,25(OH)_2_D3 and TGF-*β* individually on IL-2 production from CD4^+^ T cells have shown that they inhibit IL-2 production, which is in contrast with the data presented here where we see an enhancement of IL-2.^38^,^39^ In those studies, lower concentrations of 1,25(OH)_2_D3 were investigated, and the TGF-*β* work was performed in mice, which may in part explain the differences observed. Nevertheless, these data do provide a potential mechanism by which Foxp3^+^ T cells are increased in the presence of 1,25(OH)_2_D3 through enhanced availability of IL-2, an essential cytokine for Treg cell function, and for maintenance, survival and stability of Foxp3^+^ cells.^12^,^13^,^22^,^23^,^34^

Historically a central role in the generation of the active form of vitamin D, 1,25(OH)_2_D3, was through metabolism in the kidney from the circulating precursor 25(OH)D. However, subsequent work has shown that a number of additional cell types have the capability of generating 1,25(OH)_2_D3 from 25(OH)D, many of which are found in the lung including monocytes, dendritic cells and epithelial cells.^40^–^43^ Monocyte-derived DCs have been reported to metabolize 1,25(OH)_2_D3 to concentrations of 1 × 10^−9^ m to 6 × 10^−8^ m in culture.^42^–^44^ Hence concentrations investigated in the current study appear to approach physiologically relevant levels and probably represent concentrations potentially found in the lung. However, it is still technically challenging to address this issue experimentally. Nonetheless it seems credible that concentrations of 1,25(OH)_2_D3 required to increase Treg cell populations can be achieved locally in the tissues.

This work provides further evidence for an important role of 1,25(OH)_2_D3 as an immunomodulatory molecule, and illustrates that a wide range of concentrations of 1,25(OH)_2_D3 have the capacity to promote Foxp3^+^ Treg cells depending on the cytokine milieu. The data suggest that supplementation of vitamin-D-deficient individuals, who are reported to have reduced numbers of circulating and airway Foxp3^+^ Treg cells,^4^–^7^ may represent an attractive therapy for enhancing endogenous populations of Treg cells as seen in a recent study on subcutaneous allergen immunotherapy.^45^ Administering vitamin D supplementation to increase endogenous Treg cell populations in transplant recipients and autoimmune patients may also be an option. In addition, vitamin D treatment might provide a conditioning environment to enhance the survival and functionality of Foxp3^+^ Treg cells in strategies involving adoptive transfer of Treg cells.

In summary, 1,25(OH)_2_D3 represents an attractive approach for the treatment of chronic inflammatory diseases such as asthma. Vitamin D supplementation may offer a relatively safe, acceptable and cost-effective therapeutic option to promote peripheral tolerance and therefore warrants further investigation.

## Abbreviations

1*α*,25-dihydroxyvitamin D3: 1,25(OH)_2_D3
25-hydroxyvitamin D: 25(OH)D
IL-10: interleukin-10
STAT5: signal transducer and activator of transcription 5
TGF-*β*: transforming growth factor-*β*
Treg: regulatory T

## Supporting Information

Figure S1 Effects of treatment on viability and cell recovery.

Figure S2 1,25(OH)_2_D3 in the presence of transforming growth factor-*β* (TGF-*β*) and anti-interleukin-10 receptor antibody increases the frequency of Foxp3^+^ regulatory T cells.
