# Supplementary figures and images for: 1α,25-dihydroxyvitamin D3 in combination with transforming growth factor-β increases the frequency of Foxp3+ regulatory T cells through preferential expansion and usage of interleukin-2
Source: Immunology. 2014 Jul 29;143(1):52–60. doi: 10.1111/imm.12289 (PMC4137955; doi:10.1111/imm.12289)

**A**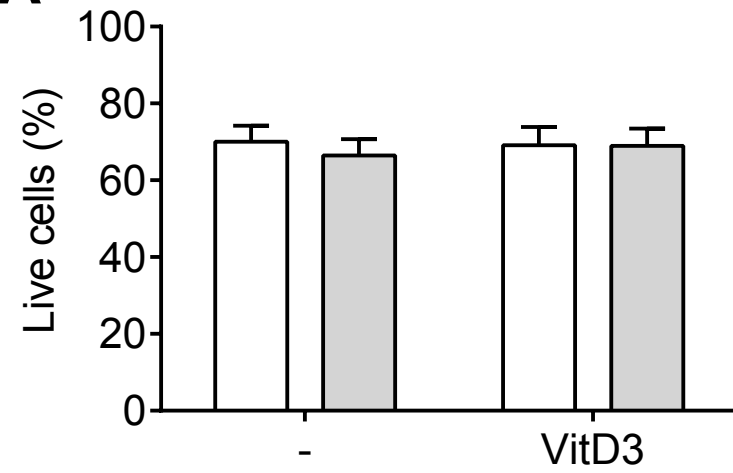

Legend:  
- (white bar)  
+ TGFβ (grey bar)

**B**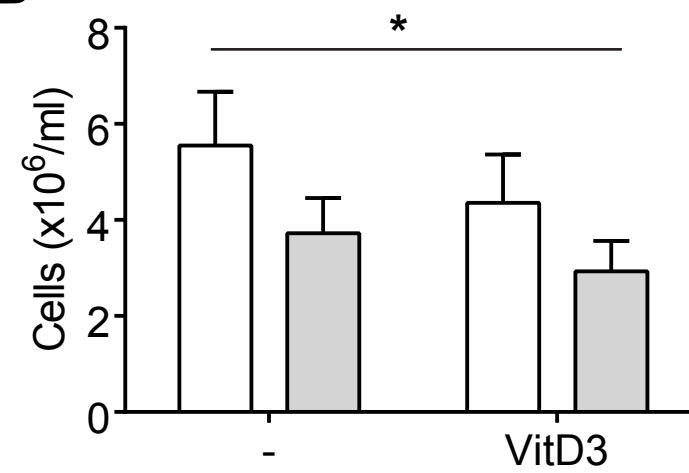

Supplement: Figure S1 — Effects of treatment on viability and cell recovery. [file imm0143-0052-sd1.pdf]

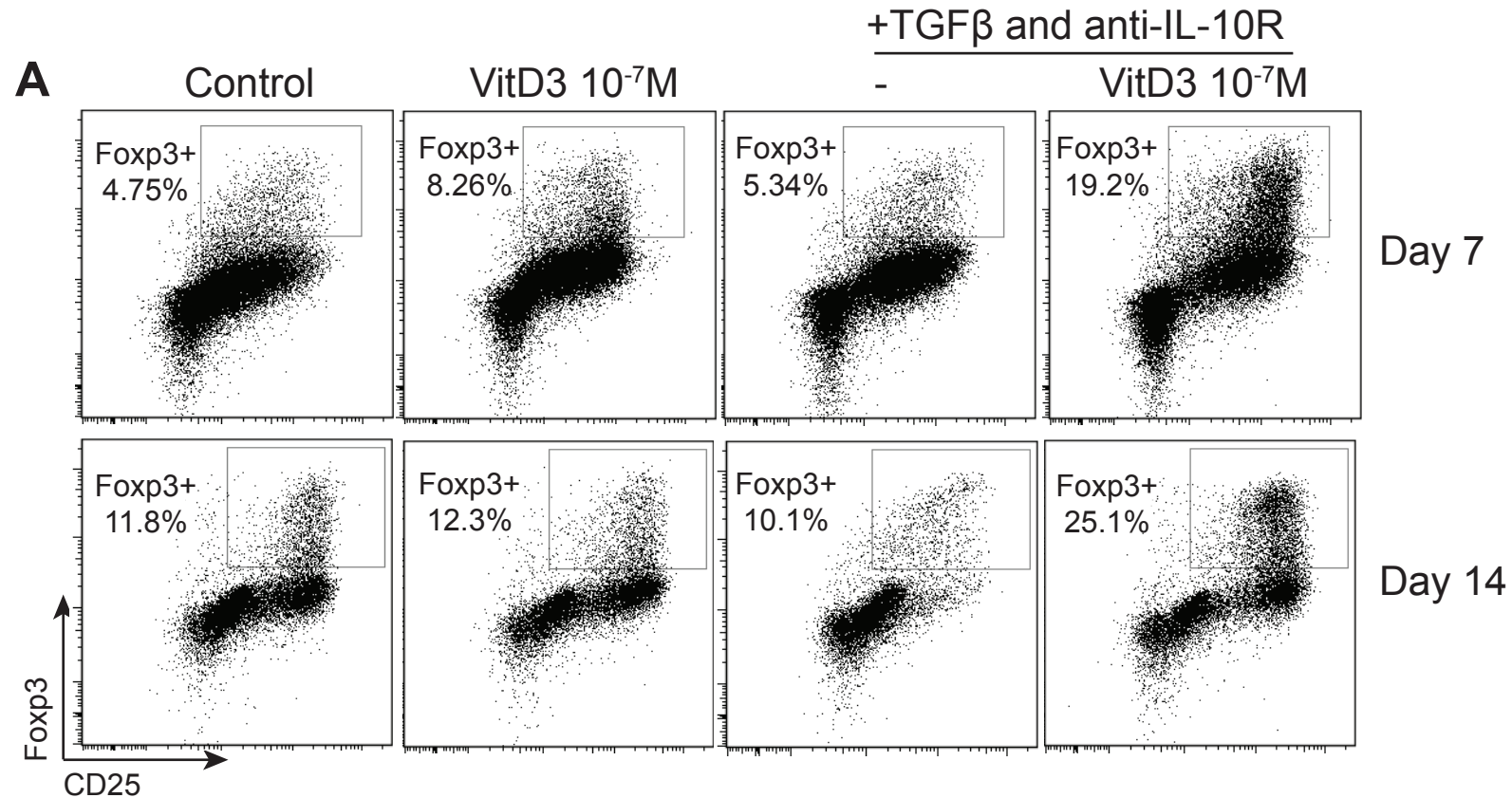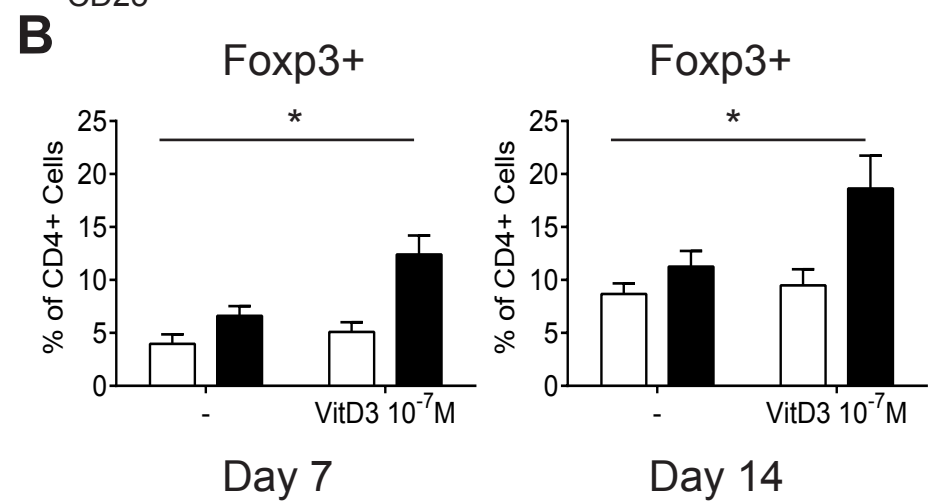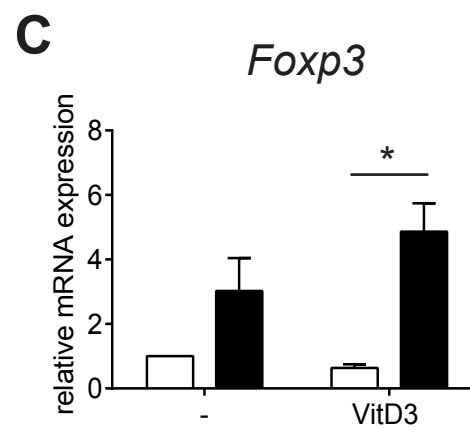

□ No Drugs

■ + TGF $\beta$  and anti-IL-10R

Supplement: Figure S2 — 1,25(OH)2D3 in the presence of transforming growth factor-β (TGF-β) and anti-interleukin-10 receptor antibody increases the frequency of Foxp3+ regulatory T cells. [file imm0143-0052-sd2.pdf]
